# Spectroelectrochemical and Theoretical Study of [Si(ttpy)_2_](PF_6_)_4_: A Potential Polychromatic Electrochromic Dye

**DOI:** 10.3390/molecules27238521

**Published:** 2022-12-03

**Authors:** Derek M. Peloquin, Askhat N. Bimukhanov, Anuar A. Aldongarov, Jon W. Merkert, Bernadette T. Donovan-Merkert, Thomas A. Schmedake

**Affiliations:** 1 Department of Chemistry, University of North Carolina at Charlotte, Charlotte, NC 28223, USA; 2 Laboratory of Physical and Quantum Chemistry, L.N. Gumilyov Eurasian National University, Astana 010008, Kazakhstan

**Keywords:** hexacoordinate silicon, spectroelectrochemistry, electrochromic, cathodic colorants, TD-DFT

## Abstract

Complexes consisting of earth-abundant main group metals such as silicon with polypyridine ligands are of interest for a variety of optical and electronic applications including as electrochromic colorants. Previous spectroelectrochemical studies with tris(2,2′-bipyridyl)silicon(IV) hexafluorophosphate, [Si(bpy)_3_](PF_6_)_4,_ demonstrated an ability to control the color saturation of the potential electrochromic dye, with the intensity of the dye’s green color increasing as the charge state sequentially reduces from 4+ to 1+. In this study, the synthesis of bis(4′-(4-tolyl)-2,2′:6′,2″-terpyridine)silicon(IV) hexafluorophosphate, [Si(ttpy)_2_](PF_6_)_4_, is reported along with electrochemical and spectroelectrochemical analyses. Computational modeling (density functional theory) is used to further elucidate the electrochromic properties of previously reported Si(bpy)_3_^n+^ species and the new Si(ttpy)_2_^n+^ species. While the homoleptic tris(bidentate)silicon(IV) complexes are attractive as electrochromic dyes for tunable color saturation, the bis(tridentate)silicon(IV) complexes are attractive as polychromatic electrochromic dyes.

## 1. Introduction

Silicon, an earth-abundant element with a strong tendency to maintain the tetravalent oxidation state, provides attractive attributes to coordination complexes for electronic and optical applications. Polypyridine ligands can bind Si(IV) to form robust and inert hexacoordinate silicon complexes capable of reversible redox chemistry with multiple stable oxidation states [1]. For example, the tris(2,2′-bipyridyl)silicon(IV) complex cation, [Si(bpy)_3_]^4+^ was shown via cyclic voltammetry to possess three chemically reversible one-electron reductions at reduction potentials significantly less negative than other bipyridine complexes due to the tetravalent nature of the silicon ion [2]. Computational modeling of [Si(bpy)]^n+^ complexes by Wieghardt and coworkers indicated silicon remains tetravalent in the reduced species with all the reductions being ligand localized [3]. Their modeling showed Si(bpy)_3_^1+^, for example, contains three ligand-localized SOMOs (≤3% Si character), and Mulliken spin density population analysis places one unpaired spin on each ligand and <0.01 on the silicon atom. It therefore has the predicted electronic structure best described as [Si^IV^(bpy˙)_3_]^+^. Furthermore, using density functional theory (DFT) with broken-symmetry they were also able to show that the previously reported black, air-sensitive compound [Si(bpy)_3_]^0^ is best described as [Si^IV^(bpy^•^)_2_(bpy^2–^)]^0^, and it possesses a singlet diradical ground state with a coupling constant *J*_calcd_ of −490 cm^–1^, which is remarkably close to the *J*_obs_ of −374 cm^–1^ [4,5].

We previously demonstrated that polypyridylsilicon(IV) complexes are attractive candidates for cathodic colorants, which are an integral component of dual-active electrochromic windows and displays [6]. In dual-active electrochromics, an electrochromic dye attached to an anode is reinforced by a complimentary cathodic electrochromic dye (cathodic colorant) [7,8,9,10]. The cathodic colorant should be colorless (or nearly colorless) in its oxidized or bleached state and intensely colored upon reduction. The *N*,*N*′-dialkyl and *N*,*N*′-diaryl-4,4′-bipyridinium salts are perhaps the best known and widely used cathodic colorants, since they possess robust and reliable spectroelectrochemistry, particularly for blue or violet coloration [11,12,13,14,15]. Polypyridyl transition metal complexes have also been evaluated as promising electrochromic dyes [16], but the metal to ligand charge transfer bands (MLCT) tend to preclude the desired colorless or bleached state. By contrast polypyridylsilicon(IV) complexes possess several advantages: (1) they exhibit multiple stable oxidation states with well-behaved chemically reversible electrochemistry, (2) they are often colorless in the fully oxidized (+4) state, (3) they have very low reduction potentials attributed to the tetravalent silicon center, which could enable low-voltage devices and applications, (4) their reduced ligand based transitions appear in the visible portion of the spectrum, and (5) they can be synthesized easily from earth-abundant elements.

In this manuscript we report the spectroelectrochemical examination of [Si(ttpy)_2_](PF_6_)_4_. We also provide an experimental and theoretical comparison of the optoelectronic properties of the bis(terpyridyl)silicon(IV) motif with the previously reported tris(bipyridyl)silicon(IV)motif and discuss their relative merits for electrochromic applications.

## 2. Results

**Scheme 1:**
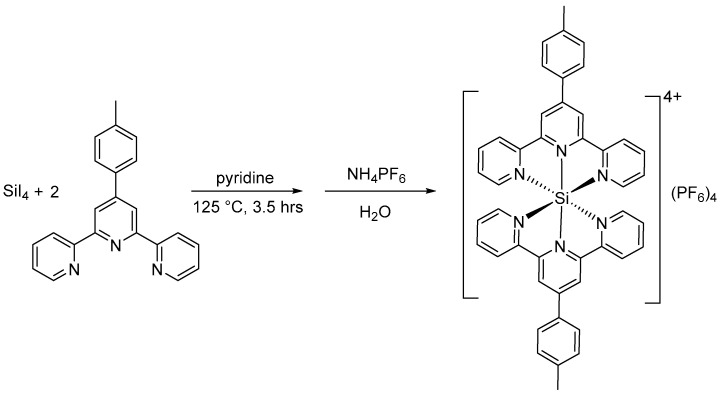
Synthesis of [Si(ttpy)_2_](PF_6_)_4_.

A precursor to the title complex, [Si(ttpy)_2_]I_4_, was synthesized by combining 4’-(p-tolyl)-2,2’:6’,2’’-terpyridine (ttpy), SiI_4,_ and pyridine in a sealed thick-walled glass pressure vessel. The reaction was heated in an oil bath behind a blast shield to 125 °C for 3.5 h. [Si(ttpy)_2_]I_4_ was converted to the hexafluorophosphate salt, [Si(ttpy)_2_](PF_6_)_4_ by metathesis with ammonium hexafluorophosphate (Scheme 1).

**Figure 1:**
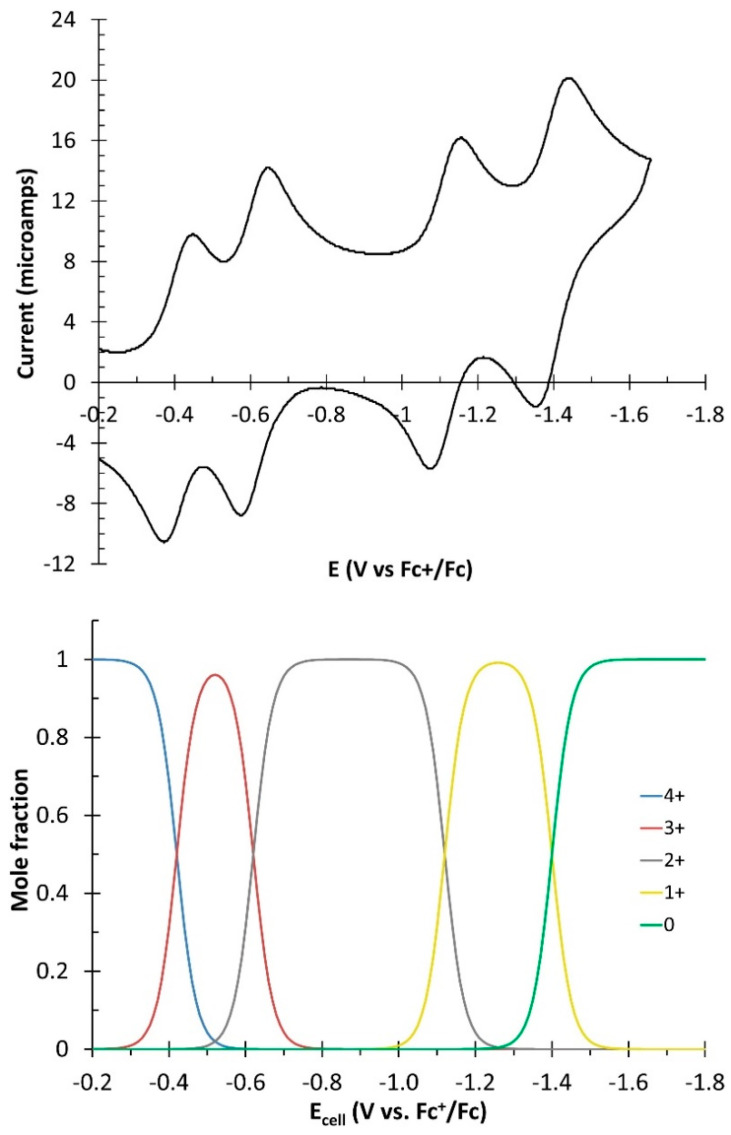
(**top**) Cyclic voltammogram of 1.0 mM [Si(ttpy)_2_](PF_6_)_4_ in acetonitrile with 0.100 M TBAPF_6_, with a platinum disk working electrode. Scan rate = 0.200 V/s with iR compensation. (**bottom**) Mole fraction of each charge state as a function of applied potential calculated from observed reduction potentials at 25 °C.

Cyclic voltammagrams of [Si(ttpy)_2_](PF_6_)_4_ in acetonitrile with tetrabutylammonium hexafluorophosphate (TBAPF_6_) as electrolyte indicated multiple stable redox states, with four chemically reversible, single electron reduction waves (Figure 1 top). The ΔE_p_ (ΔE_p_ = E_c_ − E_a_) for the waves ranged from 76 to 86 mV at a scan rate of 200 mV/s (Table 1). For comparison, previously reported values for the first four reduction waves of [Si(bpy)_3_](PF_6_)_4_ and [Si(terpy)_2_](PF_6_)_4_, where terpy is 2,2′:6′,2′′-terpyridine, are also included [2] The first reduction, $E_{1/2}^{o}$(4+/3+) = −0.403 V, is comparable to the previously reported first reduction potentials of [Si(terpy)_2_](PF_6_)_4_ and [Si(bpy)_3_](PF_6_)_4._ The low voltage required for reduction of polypyridylsilicon(IV) species is advantageous for electrochromic applications in that it allows for lower operational voltage and power demands and allows matching to low voltage anodic colorants. The difference between sequential reduction potentials is sufficient to exceed 99.9% mole fractions for the 4+, 2+, and neutral species. The 3+ and 1+ species can achieve a maximum mole fraction of 96.4% and 99.1%, respectively (Figure 1 bottom).

**Figure 2:**
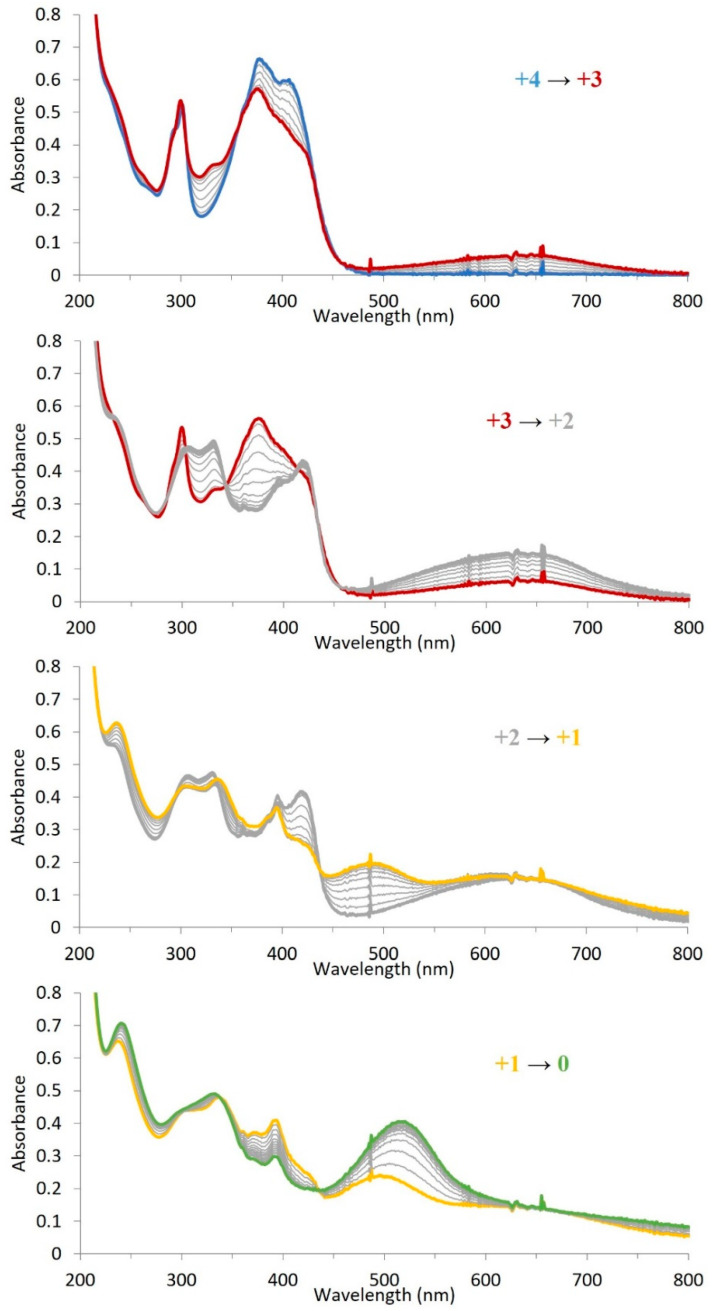
UV-vis spectra recorded at regular intervals from −250 to −525 mV (**top**), −525 to −875 (**second**), −875 to −1250 (**third**), and −1250 to −1700 mV (**bottom**).

**Figure 3:**
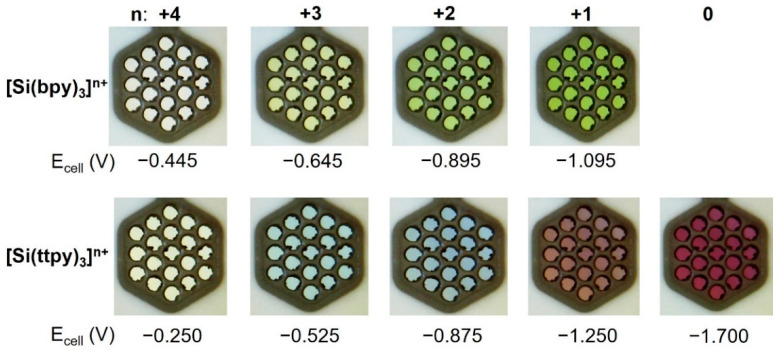
Photo of electrochromic window consisting of cathodic coloring agent [Si(bpy)_3_]^n+^ (**top row**) or [Si(ttpy)_2_]^n+^ (**bottom row**) in acetonitrile enclosed in an electrochemical cell with a gold or platinum honeycomb working electrode. Photos for [Si(bpy)_3_]^n+^ were previously reported in [6].

**Figure 4:**
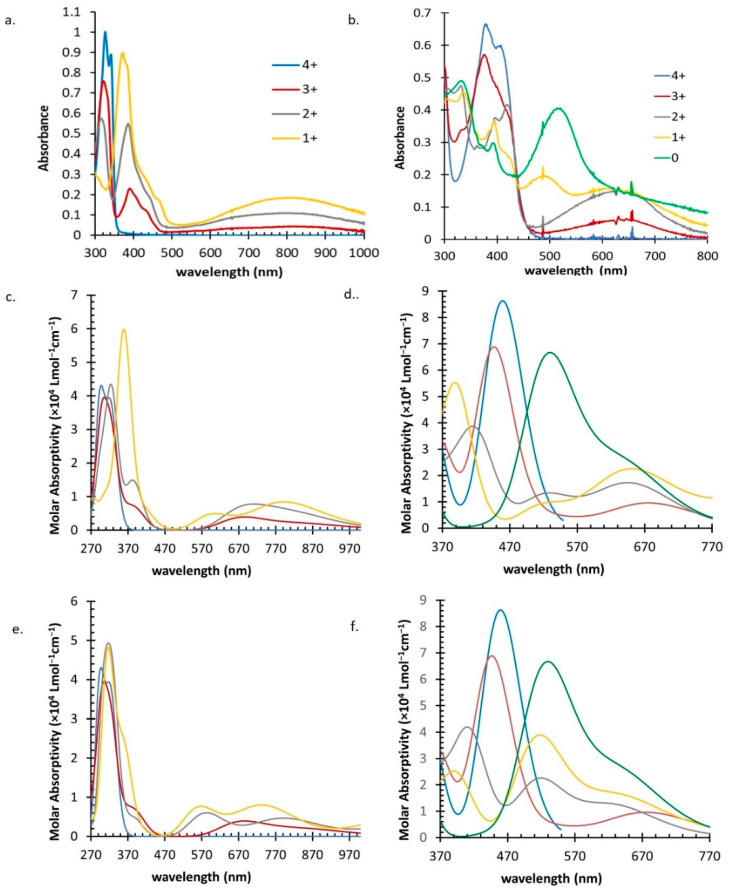
UV-vis spectra of individual charge states of (**a**) [Si(bpy)_3_]^n+^ and (**b**) [Si(ttpy)_2_]^n+^. Simulated spectra of [Si(bpy)_3_]^n+^ and [Si(ttpy)_2_]^n+^ with (**c**,**d**) high-spin ground states and (**e**,**f**) low-spin ground states, respectively. The data shown in Figure 4a were previously reported in [6].

The UV-vis spectrum of [Si(ttpy)_2_](PF_6_)_4_ in acetonitrile as a function of applied potential was obtained using an argon flushed spectroelectrochemical cell with a 1.0 mm path length that consisted of a printed gold honeycomb working electrode, a gold counter electrode, and a Ag/AgCl reference electrode standardized to the ferrocene/ferrocenium couple (Figure 2). Acetonitrile was selected as the solvent due to the low solubility of [Si(ttpy)_2_](PF_6_)_4_ in less polar solvents and the wide UV-vis and electrochemical windows acetonitrile provides. The sample was dissolved in acetonitrile with TBAPF_6_ (0.1M) as the electrolyte. Reversible transformations were observed with clean isosbestic points for each of the subsequent reductions from the 4+ state to the neutral species. The 4+ species could be recovered upon oxidation. The fully oxidized [Si(ttpy)_2_](PF_6_)_4_ has a λ_max_ = 408 nm for its lowest energy peak with a low-energy tail extending out to approximately 460 nm. Consequently, [Si(ttpy)_2_](PF_6_)_4_ has a slight yellowish appearance and not the preferred colorless appearance of [Si(bpy)_3_](PF_6_)_4_. Photos of [Si(ttpy)_2_](PF_6_)_4_ in its five separate redox states surrounded by the honeycomb electrode are provided in Figure 3 along with previously obtained photos of [Si(bpy)_3_](PF_6_)_4_ in its four chemically reversible redox states. Isolated spectra of [Si(ttpy)_2_](PF_6_)_4_ and [Si(bpy)_3_](PF_6_)_4_ for each oxidation state measured at its maximum mole fraction are included in Figure 4 along with theoretical modeling results, which are discussed later.

## 3. Discussion

Figure 3 and Figure 4 clearly demonstrate the primary difference between the tris-ligated, D_3_-symmetric [Si(bpy)_3_]^n+^ species and bis-ligated, D_2d_-symmetric [Si(ttpy)_2_]^n+^. With [Si(bpy)_3_]^n+^, the first electron goes into a ligand localized bipyridine ligand to create a bipyridine radical anion, represented as [Si^IV^(bpy)_2_(bpy^•^)]^3+^. Evidence of the bipyridine radical anion is seen in the UV-vis spectrum of [Si(bpy)_3_]^3+^ with the formation of a peak around 390 nm and a broad absorbance from 600–1000 nm (Figure 4). Subsequent reductions appear to lead to [Si^IV^(bpy) (bpy^•^)_2_]^2+^ and [Si^IV^(bpy^•^)_3_]^1+^ with a doubling and tripling of the 390 nm and 600–1000 nm bands with each additional reduction.

The net effect for electrochromic application is a voltage-tunable color intensity as seen in Figure 3 with increasing reduction leading to a more intense green color. By comparison, the [Si(ttpy)_2_]^4+^ is seen to proceed through multiple color transitions starting from a yellow species (4+), to a cyan (3+ and 2+) and finally a magenta color (1+ and neutral). The UV-vis spectrum (Figure 4b.) shows that the first reduction of [Si(ttpy)_2_]^4+^ introduces a broad peak centered around 650 nm, which we attribute to formation of a tolyl-terpyridine radical anion and a species best represented as [Si^IV^(ttpy)(ttpy^•^)]^3+^. The second electron appears to generate a second tolyl-terpyridine radical anion [Si^IV^(ttpy^•^)_2_]^2+^ with a doubling of the broad band at 650 nm. The third reduction adds a new peak around 500 nm in addition to the 650 nm peak, which are assigned to the ttpy dianion and ttpy radical anion, respectively, for [Si^IV^(ttpy^2-^) (ttpy^•^)]^1+^. The fourth reduction reduces the second ttpy radical anion, producing [Si^IV^(ttpy^2-^)_2_]^0^ consistent with the doubling of the ttpy dianion peak at 500 nm and a disappearance of the radical anion peak at 650 nm.

This explanation is consistent with a ligand-localized, “high-spin” approximation—electrons occupy separate localized ligands until forced to occupy an already reduced ligand. Due to the non-involvement of the tetravalent silicon center, we summarize the species as follows.

[Si^IV^(bpy)_3_]^4+^ → [Si^IV^(bpy)_2_(bpy^•^)]^3+^ → [Si^IV^(bpy) (bpy^•^)_2_]^2+^ → [Si^IV^(bpy^•^)_3_]^1+^

[Si^IV^(ttpy)_2_]^4+^ → [Si^IV^(ttpy)(ttpy^•^)]^3+^ → [Si^IV^(ttpy^•^)_2_]^2+^ → [Si^IV^(ttpy^2-^)(ttpy^•^)]^1+^ → [Si^IV^(ttpy^2-^)_2_]

To test this theory, DFT and TD-DFT calculations were performed using the B3LYP functional with the 6-31G* basis set. For this study, spin-restricted Kohn–Sham (RKS) solutions were obtained for closed-shell (S = 0) species and unrestricted Kohn-Sham (UKS) solutions were obtained for open shell species. Although, Wieghardt demonstrated the broken symmetry (BS) DFT approach provided spin-coupled solutions for reduced Si(bpy)_3_^n+^ species in the gas phase that were in better agreement with experimental observations, we limited our analysis in this study to the RKS and UKS solutions due to computational cost and difficulties with integrating BS-DFT with solvation and TD-DFT.

**Figure 5:**
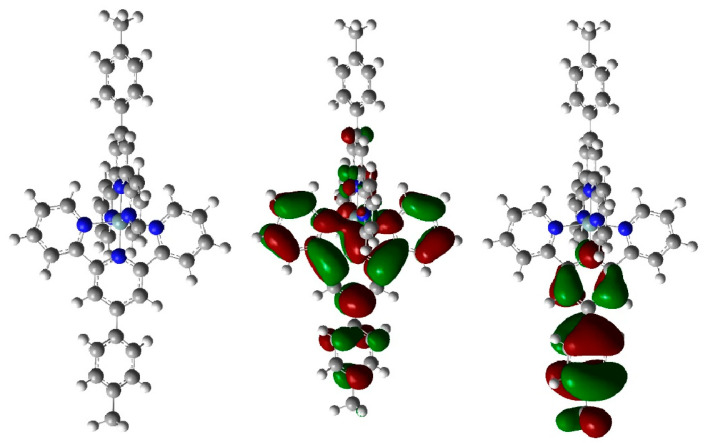
Optimized structure (**left**), HOMO (**center**), and LUMO (**right**) of [Si(ttpy)_2_]^4+^, S = 0, solvated with CH_3_CN.

Each species was optimized and confirmed to be a local minimum (no negative-frequency vibrational modes). An optimized structure of [Si(ttpy)_2_]^4+^, S = 0, with solvation (acetonitrile) along with the HOMO and LUMO of the species is shown in Figure 5. The HOMO and HOMO-1 were nearly degenerate, ΔE < 0.0001 Hartrees, with the HOMO-1 localized on the opposite ligand. Similarly, the LUMO and LUMO+1 were also nearly degenerate with ΔE < 0.0001 Hartrees. The results of all calculations are summarized in Table 2. The formatted checkpoint files for all 30 calculations are available for download in the Supplementary Materials. For both species the high-spin states were generally found to be more stable. A notable exception was for [Si(ttpy)_2_]^1+^ in the gas state, for which the low-spin state (S = 0.5) was found to be slightly more stable than the high-spin state (S = 1.5). The situation was reversed for [Si(ttpy)_2_]^1+^ when solvation effects were included though. In addition, the closed shell, low-spin (S = 0) solution is the most stable for neutral [Si(ttpy)_2_].

Simulated UV-vis structures were constructed from the RKS and UKS ground state structures using TD-DFT calculations (500 transitions, solvent = acetonitrile). Figure 4c,d shows the results using the high-spin ground states where available and the lower row (e and f) shows the results from using the low-spin ground states where available. Note that for [Si(ttpy)_2_], only the low-spin (S = 0, closed shell) ground state was included in TD-DFT calculations.

For [Si(bpy)_3_]^n+^, TD-DFT (B3LYP/6-31G*, with solvation) provides qualitative agreement between the simulated and experimental UV-vis spectra of the respective complexes when the high-spin ground state is used. Specifically, in Figure 4c, both the high energy peak around 390 nm and the broad absorbance from 600–1000 nm that are associated with the bipyridine radical anion are evident (although slightly shifted to higher energies) and they are seen to increase in intensity with increasing reductions of the overall charge. Further optimization of functionals and basis sets should improve the absolute energy alignment of these transitions. On the other hand, TD-DFT from the low-spin ground state (4e) is unsuccessful in reproducing the experimental features.

The distinction is a little less clear with Si(ttpy)_2_. The spectra of the three unambiguous species [Si(ttpy)_2_]^4+^, [Si(ttpy)_2_]^3+^, and [Si(ttpy)_2_]^0^ nicely correlate with the observed transitions in the UV-vis (although shifted in energy). With respect to [Si(ttpy)_2_]^2+^ and [Si(ttpy)_2_]^1+^, both simulated spectra appear to show peaks consistent with the experimentally observed peaks at 500 nm and 650 nm. The inability to clearly distinguish a better model between these two solutions, suggests spin-coupling may be considerable in these species and a broken symmetry BS-DFT approach may be required for optimal reproduction of the spectroelectrochemical properties for these two states.

## 4. Materials and Methods

All quantum chemical calculations were performed using density functional theory (DFT) realized in Gaussian 16 Rev. C.01 program packages [17,18,19,20]. We performed calculations applying Becke three parameter hybrid functional [21] which uses the non-local correlation provided by the Lee, Yang, and Parr expression—B3LYP [22,23]. As a basis set we have employed standard basis set 6-31G* based on a gaussian type of functions. When applicable, solvation was included using the scrf = (solvent = acetonitrile) command in Gaussian 16, which employs a Polarizable Continuum Model (PCM) [24]. The combination of functional and basis set was selected based on the success of previous attempts to model the electronic spectra of hexacoordinate silicon compounds using time-dependent DFT approach. Simulated UV-vis spectra were constructed by transforming calculated UV-vis transitions to Gaussian curves with 0.2 eV FWHM. For all calculated structures optimized structures were obtained. Frequencies calculations demonstrated absence of negative values indicating that all obtained structures correspond to the global minimum.

All reagents were used as received without further purification. SiI_4_ and 2–picoline were all stored and used under a nitrogen atmosphere in the glovebox. Acetonitrile was HPLC–grade solvent from Sigma AldrichNMR spectra were acquired using a JEOL 300 MHz NMR spectrometer and referenced to tetramethylsilane (TMS).

Cyclic voltammetry was performed using a Princeton Applied Research model 273A potentiostat/galvanostat employing a conventional three–electrode setup consisting of a platinum disk working electrode, a Ag/AgCl reference electrode, and a platinum wire auxiliary electrode. Positive feedback iR compensation was routinely used. Acetonitrile with 0.100 M TBAPF_6_ was used as the solvent and supporting electrolyte and ferrocene was used as an internal standard. Ferrocene was used as an internal standard with all potentials reported as V versus Fc/Fc+. Plots of I_pf_ vs. V^1/2^ for all reduction waves are included in the (Supplemental Materials Figures S6–S9). 

The spectroelectrochemical cell was purchased from Pine Research Instrumentation and consisted of a covered quartz cuvette with a 1.0 mm pathlength with a printed gold or platinum honeycomb working electrode, a matching (gold or platinum) counter electrode, and a Ag/AgCl reference electrode standardized to the ferrocene/ferrocenium couple (Figure S10). Samples were prepared in acetonitrile with 0.100 M TBAPF_6_ and degassed with argon that had been bubbled through acetonitrile. Potentials were then applied to the solution with a Princeton Applied Research model 173 potentiostat/galvanostat and analyzed with an Agilent 8453 Diode Array Spectrometer. UV–Vis spectra were taken in 25 mV increments with at least 45 s of equilibrium time between applied voltage values.

4′–p–tolyl–2,2′:6′,2”–terpyridine (ttpy): The ttpy ligand was synthesized following the procedure of Liu and coworkers [25]. 100 mL of ethanol was stirred in a 250 mL RBF equipped with a reflux condenser. 4.828 g (39.9 mmol) of 2–acetylpyridine, 2.431 g (20.2 mmol) of p–tolualdehyde, 1.632 g (40.8 mmol) of solid NaOH, and 65 mL concentrated aqueous ammonia were then added and stirred for 24 h at 34 °C. The resulting precipitate upon cooling was filtered and recrystallized three times from ethanol before drying under vacuum. This afforded 2.385 g of colorless crystals of ttpy in 36.4% yield. GC–MS: 323.4 m/z. ^1^H NMR (300 MHz, CD_3_CN): δ(ppm): 2.42 (s, 3 H), 7.38–7.46 (m, 4 H), 7.80 (d, 2 H), 7.92–7.98 (m, 2 H), 8.67–8.72 (m, 6 H). See Figure S1.

[Si(ttpy)_2_]I_4_: Ttpy ligand (0.244 g, 0.76 mmol) was combined with 0.16 g (0.30 mmol) of SiI4 and 3.00 mL of pyridine in a heavy walled glass pressure vessel equipped with a stir bar inside a nitrogen-filled glovebox. The vessel was transferred to an oil bath heated to 125 °C for 3.5 h. Following the reaction, the vessel was opened and 1 mL of methanol was added to quench unreacted residual iodosilanes. The contents of the vessel were transferred to a centrifuge tube, and the contents were centrifuged to afford a dark precipitate. The supernatant was decanted and the precipitate was rinsed twice with chloroform and twice with diethyl ether before drying overnight in a vacuum oven. This produced 0.302 g of [Si(ttpy)_2_]I_4_ which was used in the next step without futher purification. ^1^H NMR (300 MHz, D_2_O) δ(ppm): 2.44 (s, 6 H), 7.58 (d, J = 7.7 Hz, 4 H), 7.80–7.88 (m, 8 H), 8.24 (d, J = 8.0 Hz, 4 H), 8.62 (dd, 4 H), 9.13 (d, J = 8.3 Hz, 4 H), 9.67 (s, 4 H). See Figure S2. 

[Si(ttpy)_2_](PF_6_)_4_: 0.050 g (0.042 mmol) of [Si(ttpy)_2_]I_4_ was dissolved in 40 mL of water in a centrifuge tube. Addition of 5 mL (large excess) of saturated NH_4_PF_6_ in water caused immediate precipitation of a yellow–colored salt which was isolated by centrifugation and rinsed with deionized water two more times. ^1^H NMR (300 MHz, CD_3_CN) δ(ppm): 2.62 (s, 6 H), 7.72–7.86 (m, 8 H), 7.86 (d, *J* = 7.8 Hz, 4 H), 8.40 (d, *J* = 8.0 Hz, 4 H), 8.68 (dd, 7.8 Hz, 4 H), 9.09 (d, *J* = 7.7 Hz, 4 H), 9.54 (s, 4 H). ^13^C{^1^H} NMR (75 MHz, CD_3_CN) δ(ppm): 21.2, 124.6, 127.8, 130.1, 131.0, 131.4, 141.2, 143.9, 145.7, 147.7, 148.9. ^29^Si NMR (59.6 MHz, CD_3_CN) δ(ppm): −154. See Figures S3–S5. Anal. Calc. for C_44_H_34_F_24_N_6_P_4_Si · 2 H_2_O: C, 40.94; H, 2.97; N, 6.51. Found: C, 40.93; H, 3.12; N, 6.27%. 

## 5. Conclusions

Polypyridylsilicon(IV) complexes continue to hold promise as cathodic colorants in electrochromic applications. Due to the non-involvement of the tetravalent silicon center in the redox activity of the complex, ligand localized reductions dominate the observed spectroelectrochemical properties. Furthermore, a structure-property relationship is evident with homoleptic tris-bidentate complexes such as Si(bpy)_3_^n+^ providing a motif for voltage tunable variable color-saturation cathodic colorants and homoleptic bis-tridentate complexes such as Si(ttpy)_2_^n+^ providing a motif for voltage tunable polychromatic cathodic colorants. TD-DFT (B3LYP/6-31G*, with solvation) has been shown to accurately simulate all of the features of the UV-vis spectra of Si(bpy)_3_^n+^ throughout its positive charge states, although further benchmarking of the functionals and basis sets to better match the energy levels with observed energy levels is warranted. On the other hand, TD-DFT (B3LYP/6-31G*, with solvation) failed to accurately model some of the open-shell states of Si(ttpy)_2_^n+^ suggesting a broken symmetry DFT approach allowing for radical coupling may be necessary to model these states. Continued experimental and theoretical development of polypyridylsilicon(IV) complexes is still needed to facilitate broader electrochromic applications.

## Figures and Tables

**Table 1:** Reduction potentials for [Si(ttpy)_2_](PF_6_)_4_, [Si(terpy)_2_](PF_6_)_4_, and [Si(bpy)_3_](PF_6_)_4_.

	$E_{1/2}^{o}\;\;(V\;\mathrm{vs}.\;\mathrm{Fc}/{\mathrm{Fc}}^{+})$			
Species	4+/3+ (ΔE_p_ (mV))	3+/2+ (ΔE_p_ (mV))	2+/1+ (ΔE_p_ (mV))	1+/0 (ΔE_p_ (mV))
Si(ttpy)_2_	−0.403 (86)	−0.609 (84)	−1.111 (80)	−1.391 (76)
Si(bpy)_3_^a^	−0.530 (66)	−0.758 (66)	−1.001 (76)	−1.453 (72)
Si(terpy)_2_^a^	−0.330 (78)	−0.560 (74)	−1.102 (66)	−1.419 (80)

^a^ Previously reported data from [2].

**Table 2:** The self-consistent field calculation (SCF) reported in Hartrees for the geometry-optimized ions [Si(bpy)_3_]^n+^ and [Si(ttpy)_2_]^n+^ with charge (n) and total spin (S).

Species	n	S	SCF (Hartrees) Without CH_3_CN	SCF (Hartrees) With CH_3_CN
Si(bpy)_3_	4+	0	−1774.39697	−1775.14505
	3+	0.5	−1774.88903	−1775.31019
	2+	1	−1775.27355	−1775.46057
	2+	0	−1775.26266	−1775.45023
	1+	1.5	−1775.54027	−1775.59060
	1+	0.5	−1775.53422	−1775.58471
Si(ttpy)_2_	4+	0	−2314.06759	−2314.73001
	3+	0.5	−2314.51651	−2314.89705
	2+	1	−2314.87754	−2315.05197
	2+	0	−2314.86724	−2315.04067
	1+	1.5	−2315.12118	−2315.17060
	1+	0.5	−2315.12151	−2315.16962
	0	2	−2315.25913	−2315.27181
	0	1	−2315.26448	−2315.27772
	0	0	−2315.26866	−2315.28250

## Data Availability

All data are fully available and included in the manuscript.

## Supplementary Materials

The following supporting information can be downloaded at: https://www.mdpi.com/article/10.3390/molecules27238521/s1, Figures S1–S5: NMR data. Figures S6–S10: electrochemical and spectroelectrochemical data and device schematic. Computational methods details. Formatted checkpoint files for all 30 species included in Table 2.

## Sample Availability

Samples of the compounds are not available.

## References

[1] Peloquin D.M., Schmedake T.A. (2016). Recent Advances In Hexacoordinate Silicon With Pyridine-Containing Ligands: Chemistry And Emerging Applications. Coord. Chem. Rev..

[2] Suthar B., Aldongarov A., Irgibaeva I.S., Moazzen M., Donovan-Merkert B.T., Merkert J.W., Schmedake T.A. (2012). Electrochemical And Spectral Properties of Hexacoordinate Polypyridyl Silicon Complexes. Polyhedron.

[3] England J., Wieghardt K. (2013). 2,2′-Bipyridine Compounds of Group 14 Elements: A Density Functional Theory Study. Inorg. Chem..

[4] Herzog S., Krebs F. (1963). Uber Die Erstmalige Darstellung Von Tris-2,2′-Dipyridyl-Silicium(0) Si(dipy)_3_ [On The Presentation Of Tris-2,2’-Dipyridylsilicon(0) Si(dipy)_3_]. Naturwissenschaften.

[5] Wulf E., Herzog S. (1972). Die Temperaturabhängigkeit Der Magnetischen Suszeptibilität Einiger Elementdipyridyle [Temperature Dependence Of Magnetic Susceptibility of Some Element Dipyridyls]. Z. Fur Anorg. Und Allg. Chem..

[6] Peloquin D.M., Dewitt D.R., Patel S.S., Merkert J.W., Donovan-Merkert B.T., Schmedake T.A. (2015). Spectroelectrochemistry Of Tris(bipyridyl)silicon(IV): Ligand Localized Reductions with Potential Electrochromic Applications. Dalton Trans..

[7] Beaujuge P.M., Amb C.M., Reynolds J.R. (2010). Spectral Engineering in pi-Conjugated Polymers with Intramolecular Donor-Acceptor Interactions. Acc. Chem. Res..

[8] Bulloch R.H., Kerszulis J.A., Dyer A.L., Reynolds J.R. (2014). Mapping the Broad CMY Subtractive Primary Color Gamut Using a Dual-Active Electrochromic Device. ACS Appl. Mater. Interfaces.

[9] Jensen J., Hosel M., Dyer A.L., Krebs F.C. (2015). Development and Manufacture of Polymer-Based Electrochromic Devices. Adv. Funct. Mater..

[10] Thakur V.K., Ding G.Q., Ma J., Lee P.S., Lu X.H. (2012). Hybrid Materials and Polymer Electrolytes for Electrochromic Device Applications. Adv. Mater..

[11] Madasamy K., Velayutham D., Suryanarayanan V., Kathiresan M., Ho K.C. (2019). Viologen-Based Electrochromic Materials and Devices. J. Mater. Chem. C.

[12] Mortimer R.J., Varley T.S. (2011). Novel Color-Reinforcing Electrochromic Device Based on Surface-Confined Ruthenium Purple and Solution-Phase Methyl Viologen. Chem. Mater..

[13] Mortimer R.J., Varley T.S. (2012). In Situ Spectroelectrochemistry and Colour Measurement of A Complementary Electrochromic Device Based on Surface-Confined Prussian Blue and Aqueous Solution-Phase Methyl Viologen. Sol. Energy Mater. Sol. Cells.

[14] Papadakis R. (2020). Mono- and Di-Quaternized 4,4′-Bipyridine Derivatives as Key Building Blocks for Medium- and Environment-Responsive Compounds and Materials. Molecules.

[15] Shah K.W., Wang S.X., Soo D.X.Y., Xu J.W. (2019). Viologen-Based Electrochromic Materials: From Small Molecules, Polymers and Composites to Their Applications. Polymers.

[16] Mortimer R.J. (2011). Electrochromic Materials. Annu. Rev. Mater. Res..

[17] Parr R.G., Yang W. (1989). Density-Functional Theory of Atoms and Molecules.

[18] Salahub D.R., Zerner M.C. (1989). The Challenge of d and f Electrons.

[19] Kohn W., Sham L.J. (1965). Self-Consistent Equations Including Exchange and Correlation Effects. Phys. Rev..

[20] Hohenberg P., Kohn W. (1964). Inhomogeneous Electron Gas. Phys. Rev..

[21] Becke A.D. (1993). Density-functional thermochemistry. III. The role of exact exchange. J. Chem. Phys..

[22] Lee C., Yang W., Parr R.G. (1988). Development of the Colle-Salvetti correlation-energy formula into a functional of the electron density. Phys. Rev. B.

[23] Miehlich B., Savin A., Stoll H., Preuss H. (1989). Results obtained with the correlation-energy density functionals of Becke and Lee, Yang and Parr. Chem. Phys. Lett..

[24] Tomasi J., Mennucci B., Cammi R. (2005). Quantum mechanical continuum solvation models. Chem. Rev..

[25] Liu X., Xu J., Lv Y., Wu W., Liu W., Tang Y. (2013). An ATP–Selective, Lanthanide Complex Luminescent Probe. Dalton Trans..

