# Supplementary material for: Spectroelectrochemical and Theoretical Study of [Si(ttpy)2](PF6)4: A Potential Polychromatic Electrochromic Dye
Source: Molecules. 2022 Dec 3;27(23):8521. doi: 10.3390/molecules27238521 (PMC9738546; doi:10.3390/molecules27238521)
Supplement: Supplementary file 1 [file molecules-27-08521-s001.zip › molecules-2068328-supplementary.pdf]

Supplemental Materials

Spectroelectrochemical and Theoretical Study of $[Si(ttpy)_2](PF_6)_4$: A Potential Polychromatic Electrochromic Dye

Derek M. Peloquin [1], Askhat N. Bimukhanov [2], Anuar A. Aldongarov [2], Jon W. Merkert [1], Bernadette T. Donovan-Merkert [1] and Thomas A. Schmedake [1,*]

1 Department of Chemistry, University of North Carolina at Charlotte, Charlotte, NC 28223, USA
2 Laboratory of Physical and Quantum Chemistry, L.N. Gumilyov Eurasian National University, Astana 010008, Kazakhstan
* Correspondence: tschmeda@uncc.edu; Tel.: +1-704-687-5177

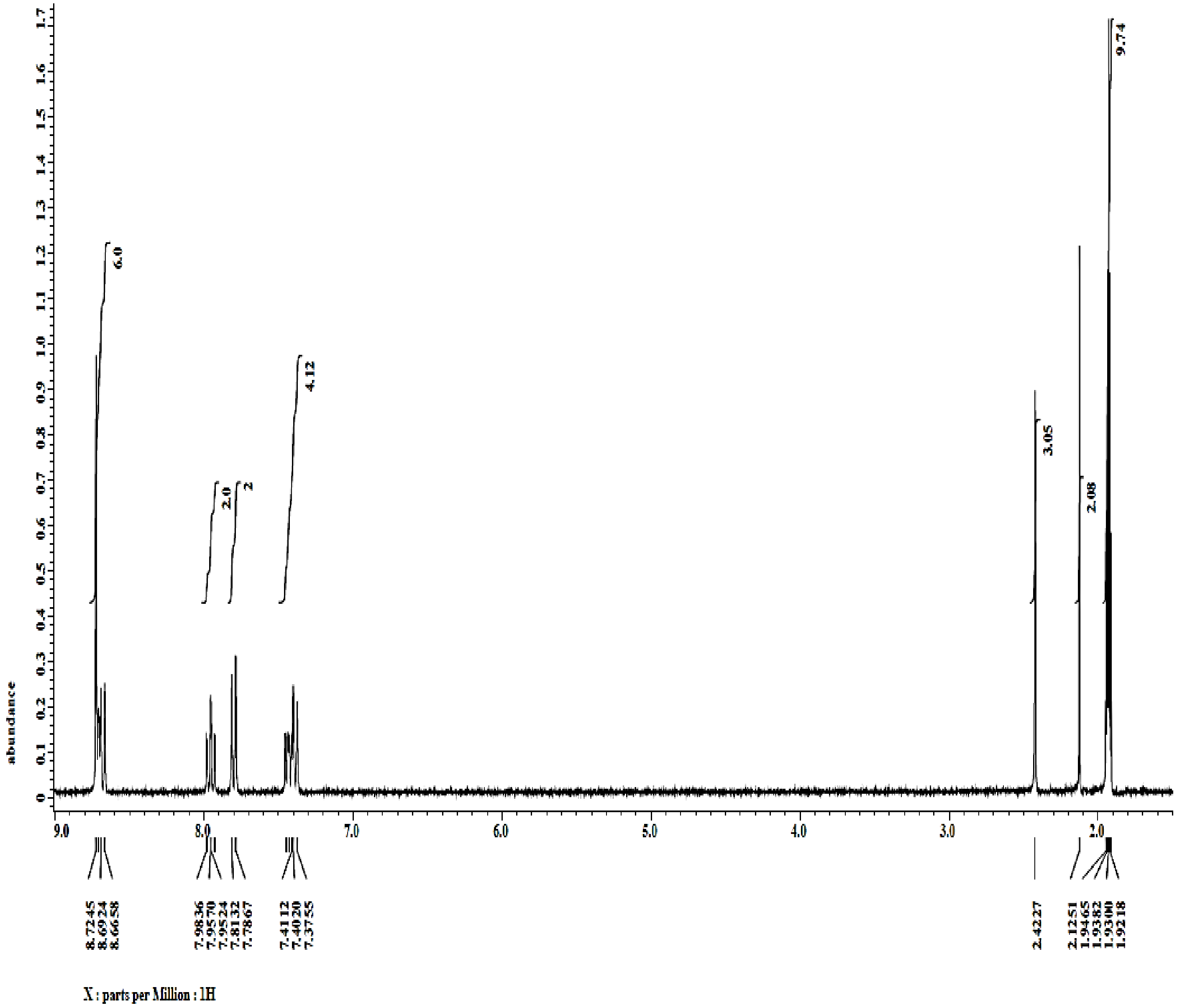

Figure S1: ^{1}H NMR spectrum of tolylterpyridine in CD_3CN.

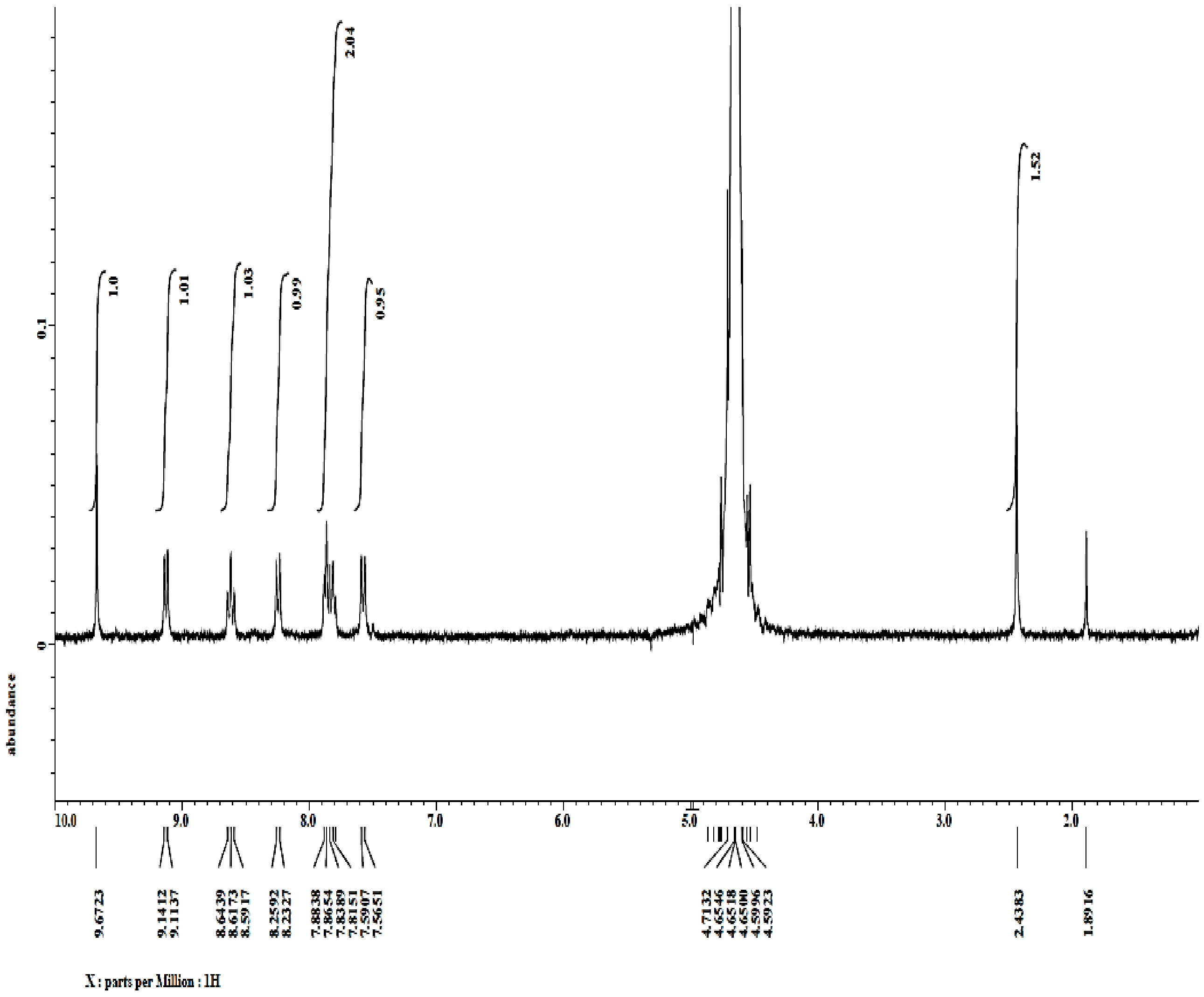

Figure S2: ^{1}H NMR spectrum of $[Si(tpy)_2]I_4$ in D_2O.

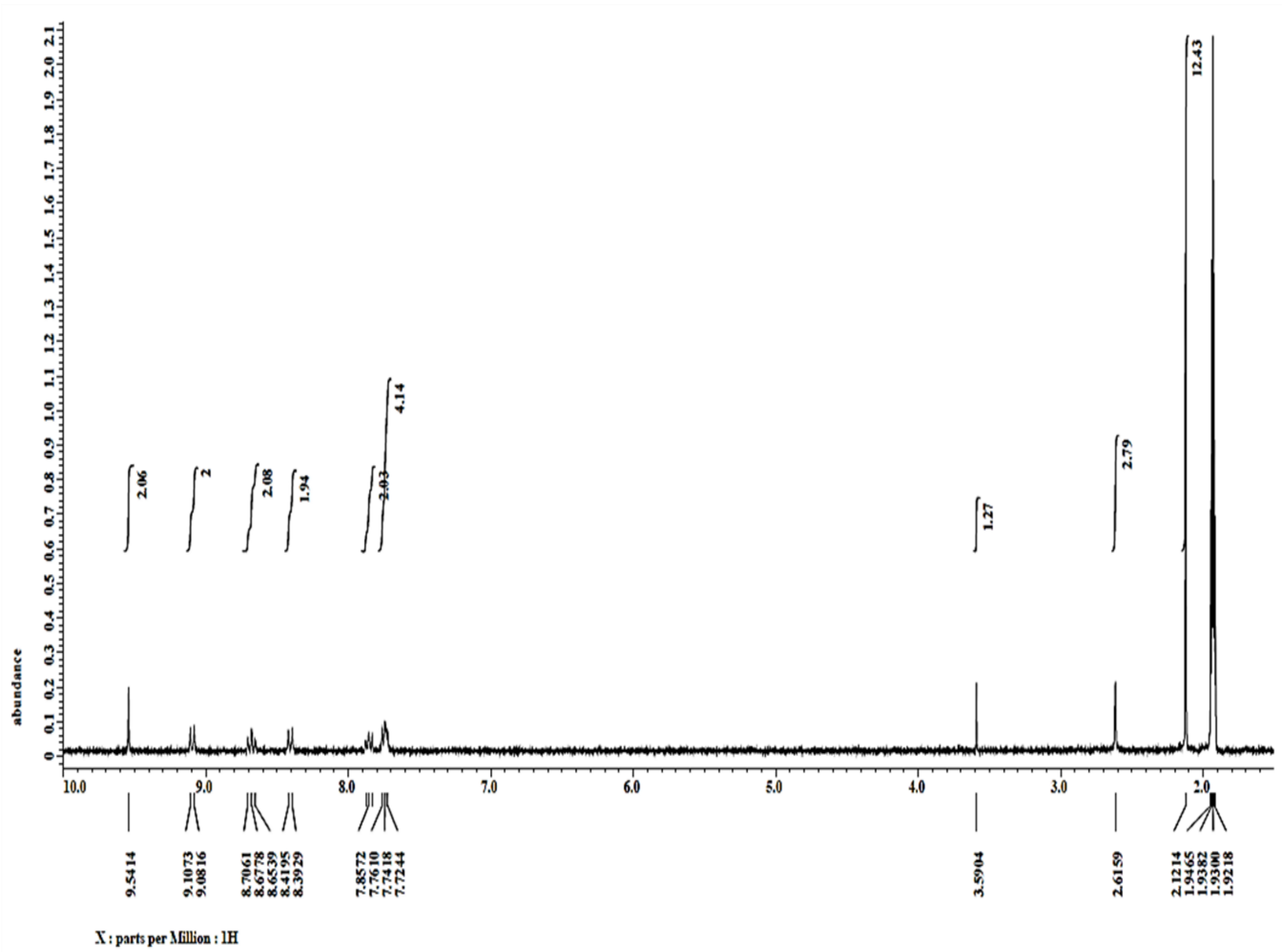

Figure S3: ^{1}H NMR spectrum of $[Si(ttpy)_2](PF_6)_4$ in CD_3CN.

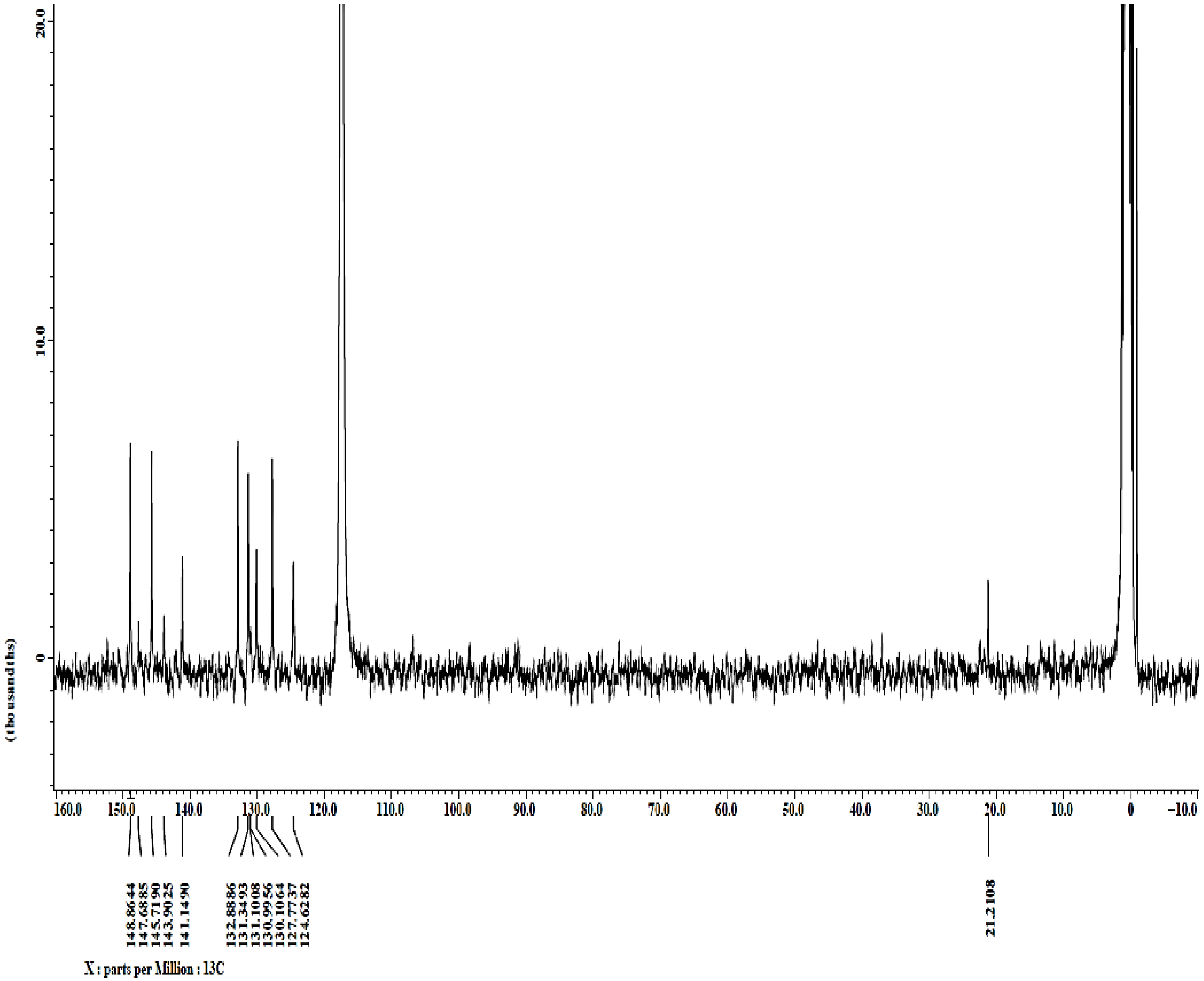

Figure S4: $^{13}C\{^{1}H\}$ NMR spectrum of $[Si(ttpy)2](PF6)4$ in CD3CN.

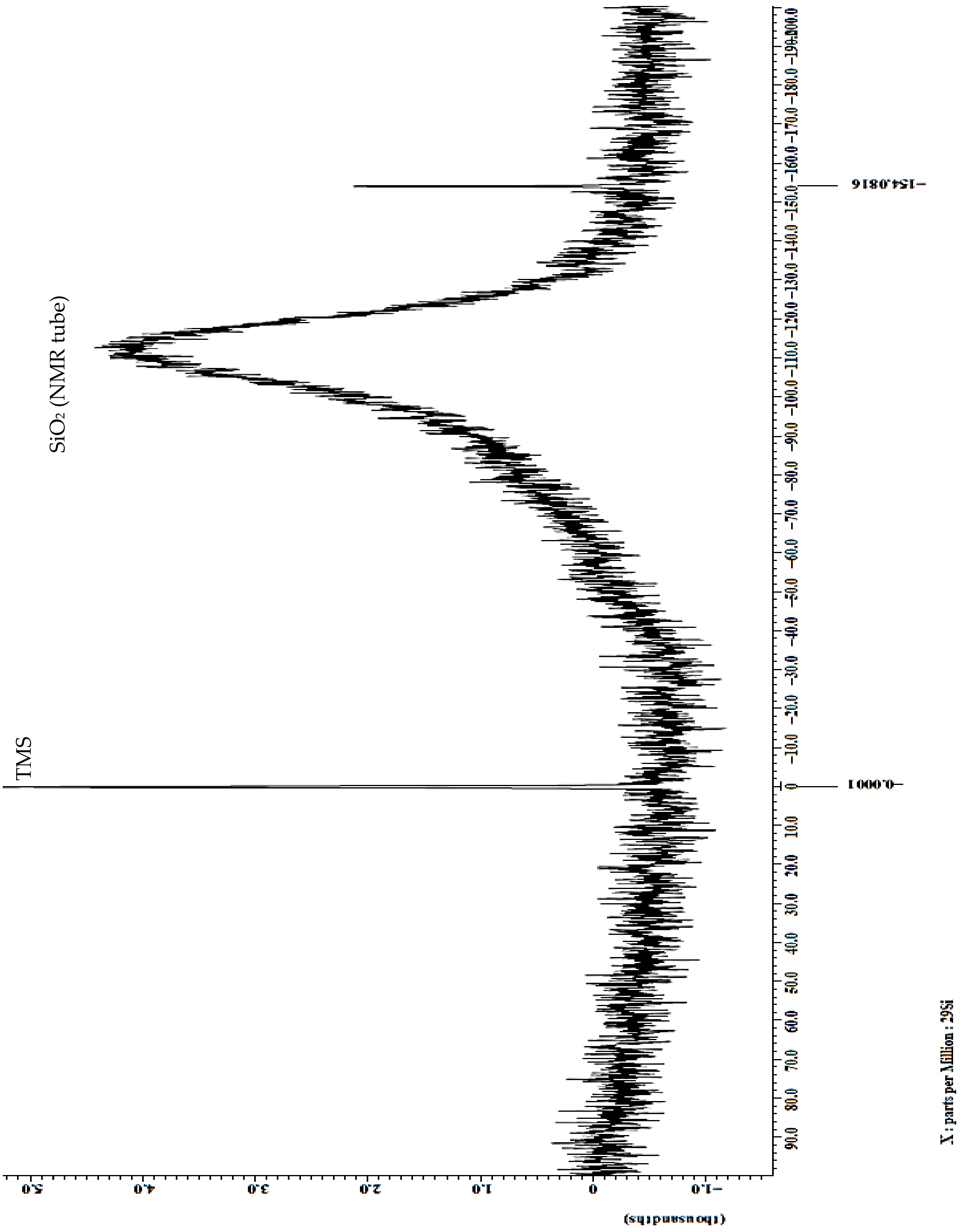

Figure S5: ^{29}Si NMR spectrum of $[Si(ttpy)_2](PF_6)_4$ with 0.05 M $Cr(acac)_3$ as a relaxation agent and tetramethylsilane as a standard in CD_3CN.

Plots of I_{pf} vs. $V^{1/2}$ for each of the 4 waves were constructed for the sweep rates 100, 200, 400, 600, and 800 mV/s. All data were consistent with the Randles-Sevcik equation over this range indicating the electrochemistry was diffusion controlled.

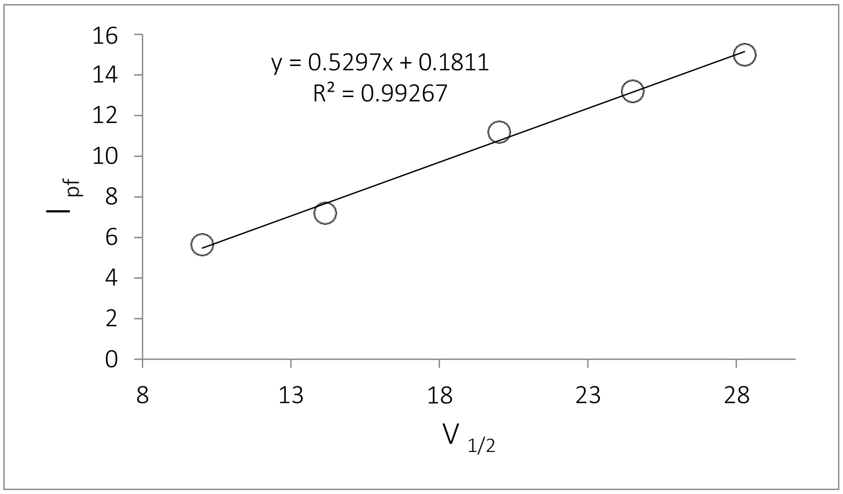

Figure S6: Plot of I_{pf} vs. $V^{1/2}$ for 4+/3+ reduction wave.

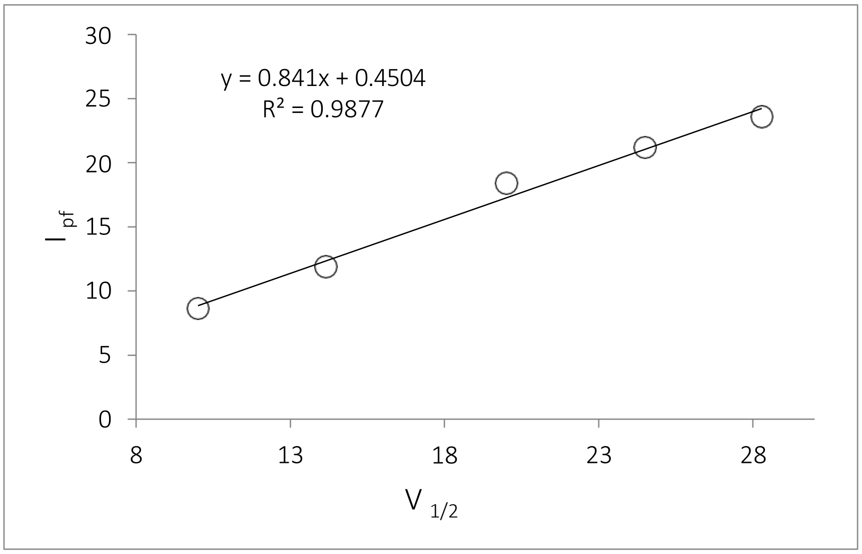

Figure S7: Plot of I_{pf} vs. $V^{1/2}$ for 3+/2+ reduction wave.

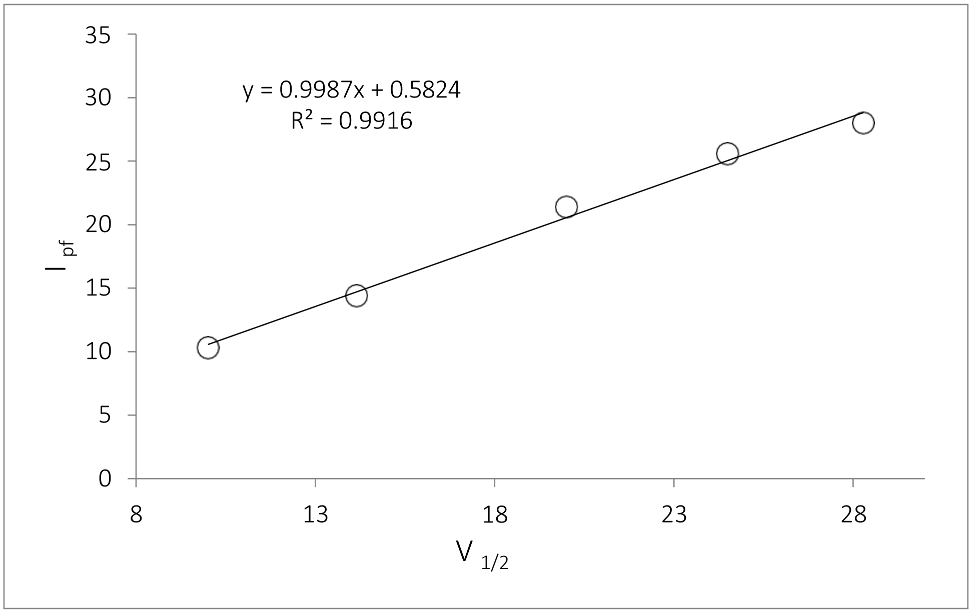

Figure S8: Plot of I_{pf} vs. $V^{1/2}$ for 2+/1+ reduction wave.

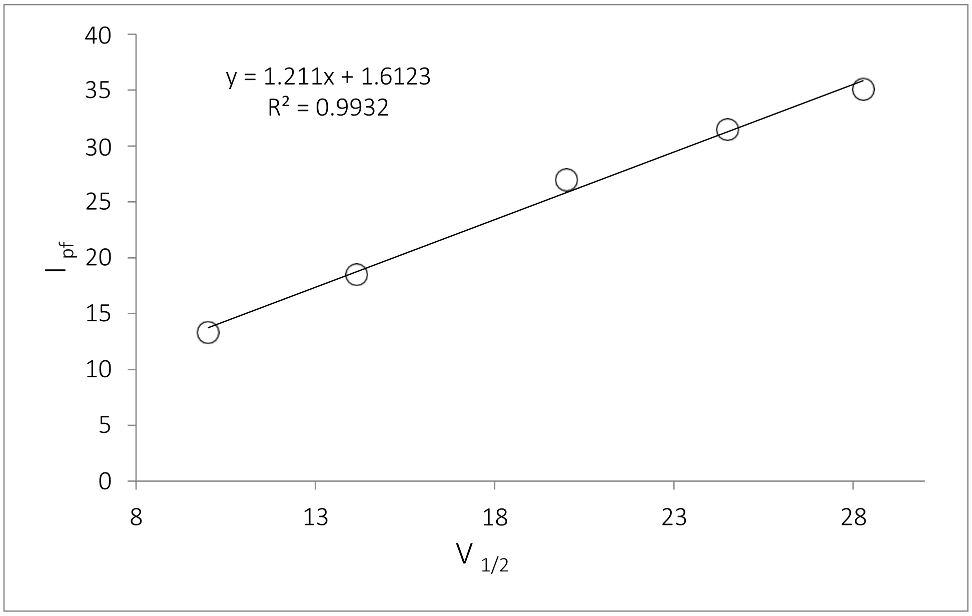

Figure S9: Plot of I_{pf} vs. $V^{1/2}$ for 1+/0 reduction wave.

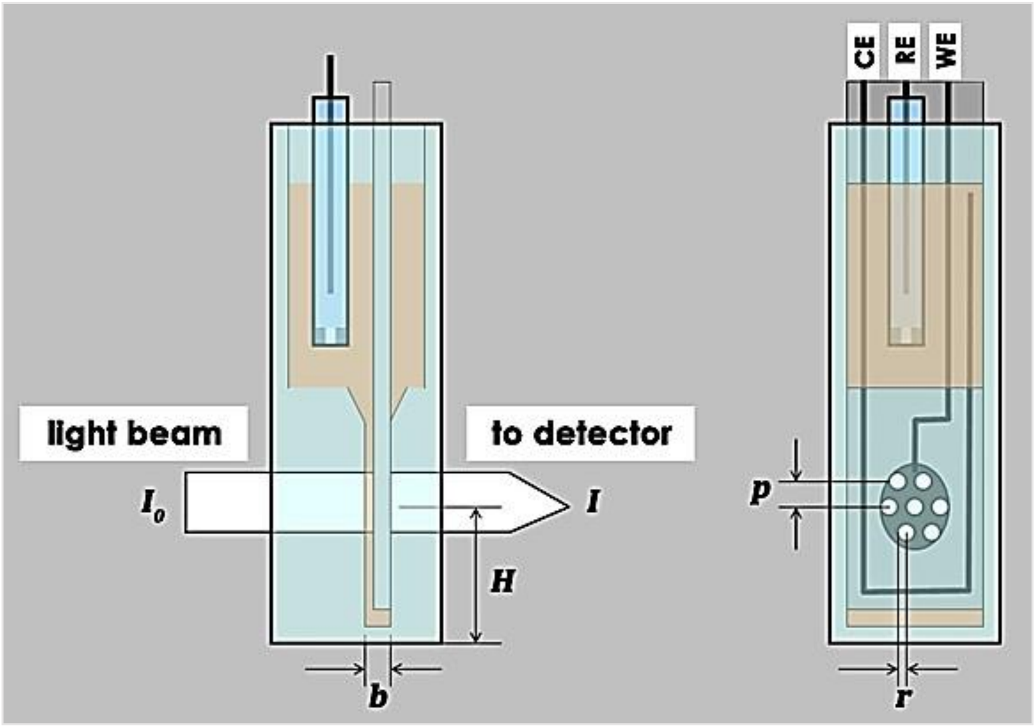

Figure S10. Schematic of spectroelectrochemical cell.

From: "Honeycomb spectroelectrochemical cell user guide." Pine Research Instrumentation: Durham, NC, 2013.

Computational Methods:

All quantum chemical calculations were performed using density functional theory (DFT)[i] realized in Gaussian 16 Rev. C.01 program packages. We have performed calculations applying Becke three parameter hybrid functional[ii] which uses the non-local correlation provided by the Lee, Yang, and Parr expression[iii] - B3LYP. As a basis set we have employed standard basis set 6-31G* based on a gaussian type of functions. The combination of functional and basis set was selected based on precision of prediction of the electronic spectrum of silicon hexacoordinated compound using time-dependent DFT approach. Simulated UV-vis spectra were constructed by transforming calculated UV-vis transitions to Gaussian curves with 0.2 eV FWHM.

For all calculated structures optimized structures were obtained. Frequencies calculations demonstrated absence of negative values indicating that all obtained structures correspond to the global minimum. Sample job commands are included below.

Geometric optimization and vibrational frequency calculations:

```
# b3lyp/6-31g* Opt freq scrf=(solvent=acetonitrile) geom=connectivity
```

TD-DFT calculations:

```
# b3lyp/6-31g* TD(NStates=500) scrf=(solvent=acetonitrile)
```

---

[i] (a) R. G. Parr and W. Yang, Density-functional theory of atoms and molecules (Oxford Univ. Press, Oxford, 1989) (b) The Challenge of d and f Electrons, Ed. D. R. Salahub and M. C. Zerner (ACS, Washington, D.C., 1989). (c) W. Kohn and L. J. Sham, “Self-Consistent Equations Including Exchange and Correlation Effects,” Phys. Rev., 140 (1965) A1133-A38. (d) P. Hohenberg and W. Kohn, “Inhomogeneous Electron Gas,” Phys. Rev., 136 (1964) B864-B71.

[ii] A. D. Becke, “Density-functional thermochemistry. III. The role of exact exchange,” J. Chem. Phys., 98 (1993) 5648-52.

[iii] (a) C. Lee, W. Yang, and R. G. Parr, "Development of the Colle-Salvetti correlation-energy formula into a functional of the electron density," Phys. Rev. B, 37 (1988) 785-89. (b) B. Miehlich, A. Savin, H. Stoll, and H. Preuss, "Results obtained with the correlation-energy density functionals of Becke and Lee, Yang and Parr," Chem. Phys. Lett., 157 (1989) 200-06.
